# Study on the Properties and Design Applications of Polyester–Cotton Matrix Mycelium Composite Materials

**DOI:** 10.3390/biomimetics10100681

**Published:** 2025-10-10

**Authors:** Wanlin Zheng, Yajie Gao, Xiaona Zong, Jun Wang

**Affiliations:** 1 School of Fashion, Dalian Polytechnic University, Dalian 116034, China; 18686481770@163.com (W.Z.); gaoyajie@dlpu.edu.cn (Y.G.); z18525348389@163.com (X.Z.); 2 National Demonstration Center for Experimental Fashion Design and Engineering Education, Dalian Polytechnic University, Dalian 116034, China

**Keywords:** polyester–cotton matrix, mycelium composite material, applied performance, experiential home products, sustainable design

## Abstract

The increased consumption of apparel has resulted in a corresponding increase in the volume of waste textiles, with polyester–cotton blended textiles accounting for as much as 80% of the total. However, extant recycling methodologies are beset by challenges, including high cost and difficulty in separation. Mycelium has been shown to possess the ability to degrade complex components in culture substrates. The present study explores the feasibility of using polyester–cotton yarn as a substrate for mycelium composite materials, thus offering an innovative approach to the treatment of waste blended textiles. Five mycelium composite materials with varying polyester–cotton ratios were prepared and tested for mechanical strength, moisture resistance, and biodegradability. ANOVA analysis confirmed that all properties of the mycelium composites were significantly influenced by the polyester–cotton matrix ratio, with partial eta-squared (ηp^2^) exceeding 84% across all properties. The most significant effect was observed in compressive strength (ηp^2^ > 99%). Experiments identified a 65:35 polyester–cotton ratio as yielding optimal comprehensive properties: namely, a compressive strength of 0.221 MPa and flexural strength of 0.791 MPa, coupled with excellent moisture resistance and biodegradability. This provides data support for the development of textile-based mycelium composite products. In light of the aforementioned performance studies and material characteristics, the development of three series of experiential home products was undertaken. Design evaluations were conducted to explore the potential application of mycelium composites, which could have significant implications for promoting sustainable development in the textile and apparel industry and advancing innovative designs for mycelium composite materials.

## 1. Introduction

With the continuous development of the economy, people’s demand for clothing consumption has gradually increased, leading to a surge in the volume of waste textiles and exacerbating environmental pollution. The latest 2024 United Nations report indicates that globally, 140 million tonnes of textile waste are generated annually, yet recycling rates remain exceptionally low [1]. Polyester-blended textiles constitute the largest proportion of blended fabrics [2,3], encompassing polyester–cotton blends or polyester mixed with other fibres, with polyester–cotton blends accounting for approximately 70% of this category. Consequently, research into reusing polyester–cotton blended fibres holds significant environmental importance. Common waste textile treatment methods include energy recovery [4], mechanical recycling [5,6], and chemical recycling [7,8], though these face challenges such as high recovery costs and difficulties in achieving single-component identification and separation. Thus, the rational utilisation of waste textiles represents an urgent issue requiring resolution.

Mycelium-based composites (MBCs) represent an eco-friendly material formed by integrating fungal mycelium with agricultural and textile industry waste substrates [9,10], exhibiting both biodegradability [11,12] and robust mechanical properties [13]. Research indicates that fungi can decompose compounds within the substrate, accelerating the degradation of components that would otherwise resist natural breakdown [14,15,16]. Consequently, scholars have proposed utilising textile fibres as the substrate for mycelium-based composites, investigating the properties of textile-based mycelium composites to achieve sustainable development in the apparel sector through biotechnology [17,18,19]. Waste textiles serve as a substrate for mycelium composites in two primary forms: waste textile fibres and discarded textiles. Mycelium can either composite scattered waste fibres or reinforce and repair discarded textiles through mycelial reinforcement. Qiu et al. [19] prepared mycelium composites using cotton, polyester, and polypropylene fibres. Cotton fibres exhibited superior mechanical properties, achieving a compressive strength of 5.86 MPa and a flexural strength of 5.72 MPa. Testing revealed that longer fibres enhanced the material’s flexural performance. Mycelium composites fabricated from single-fibre matrices exhibited relatively weak properties. Incorporating tobacco stalk skeleton material as reinforcement increased flexural performance by 40%, rendering the material suitable for applications in packaging and construction [18]. Huffman et al. [20] utilised mycelium to repair sports shoes. A sole mould was constructed via 3D printing, into which the culture substrate was poured. The disinfected shoe was then placed atop the mould. After two to three weeks of cultivation, the mycelium fully integrated with the shoe, achieving repair and reinforcement. Saini et al. [21] inoculated waste cotton and polyester textiles with Lentinula edodes mycelium to investigate fungal growth potential on discarded textiles. During cultivation, the degradation of textile quality was observed, indicating the fungi’s capacity to decompose textiles. Post-growth compression testing of the waste textile–mycelium composites revealed compressive strengths of 270 kPa for cotton-based composites and 100 kPa for polyester-based composites, demonstrating potential as polystyrene foam packaging substitutes. Current research on waste fibre-based mycelium composites predominantly utilises single-fibre types [18], with limited studies on multi-fibre compositions. Furthermore, material performance testing has primarily focused on mechanical properties, while research into other application-specific characteristics remains scarce.

In summary, this paper will focus on investigating the feasibility of polyester–cotton blended materials as a substrate for mycelium composite cultivation. Five substrate combinations with varying polyester–cotton ratios were selected. Samples were prepared using mould-free 3D bioprinting techniques [22,23] to minimise material wastage during fabrication [24,25,26,27], thereby offering an innovative approach to textile waste treatment. The prepared samples underwent mechanical property, moisture resistance, and biodegradability testing. ANOVA analysis quantitatively assessed the effect size of polyester–cotton ratios on material properties, determining the statistical significance of performance variations across different ratios. Finally, by leveraging the performance characteristics and unique growth properties of mycelium composites, a series of experiential mycelium composite home products were developed. This exploration aims to unlock the application potential of mycelium composites, providing theoretical references for their product design and development.

## 2. Materials and Methods

### 2.1. Experimental Materials and Equipment

The experimental materials were categorised into four types: fungal strains, polyester–cotton yarn, auxiliary culture substrates, and gelatine. The grey oyster mushroom (*Pleurotus eryngii*) mother strain, sourced from the China Minyuan Mushroom Industry, was selected as the experimental fungal strain. Oyster mushrooms have been found to possess the ability to decompose lignin and other plant components, exhibit strong adaptability to cultivation environments, and demonstrate rapid growth rates. As demonstrated in previous studies, mycelium composites prepared from grey oyster mushroom strains have been shown to exhibit excellent mechanical properties, alongside rapid growth rates [28,29,30]. Textile-type culture substrates comprised 100% polyester and 100% cotton yarn, both sourced from Jiangsu Jiangsheng Garment Accessories Co., Ltd., Changshu City, Suzhou City, Jiangsu Province, China. Waste agricultural materials serving as auxiliary substrates included wood chips [31], wheat bran, and cottonseed hulls, supplied by Jingmen Jingnong Fungus Co., Ltd., Hubei Province, China. Additionally, small quantities of sugar and lime powder [29] were incorporated into the experimental materials to enhance mycelial growth. To facilitate smoother printing, xanthan gum and carrageenan [26,27,30], sourced from Zhejiang Tianhe Food Biotechnology Co., Ltd., China, were added to improve extrusion fluidity.

The experimental apparatus comprises six categories: grinding, sterilisation, inoculation, printing, cultivation, and drying, as detailed in Table 1.

### 2.2. Material Preparation

Mycelium growth requires abundant carbon and nitrogen sources for nourishment; therefore, a certain amount of crop residue substrate was selected and added to co-cultivate the mycelium composite material with yarn substrate. In this experiment, the crop residue substrate was initially prepared in equal proportions, followed by the addition of polyester–cotton yarn substrate at varying concentrations, according to subsequent experimental designs. The composition of the crop substrate was as follows: 68% cottonseed hulls, 15% wheat bran, and 15% corn cobs. This substrate was enriched with 1% sugar and 1% gypsum powder to enhance its nutritional content.

**Figure 1:**
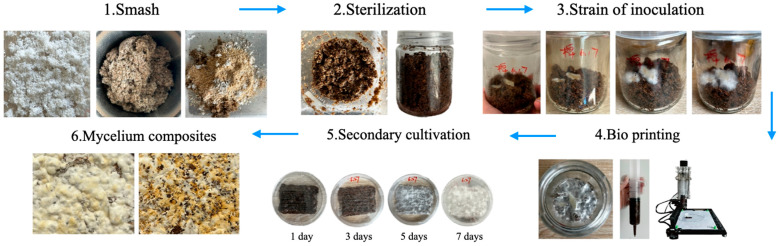
Process for preparing waste spinning-based mycelium composites.

The preparation of the mycelium composite material followed the direct extrusion 3D bioprinting method described in Alima et al.’s research [32], comprising six steps, as illustrated in Figure 1. Both the crop substrate and the yarn substrate were subjected to a grinding process, resulting in the production of uniform granular particles and short fibres measuring 1 mm, respectively. This procedure was implemented to ensure the consistency of particle size, thereby facilitating efficient mixing and extrusion processes. Subsequently, the agricultural substrate was meticulously weighed according to the previously mentioned ratio and immersed in a mixed aqueous solution containing 1% sucrose and 1% gypsum powder. The solution was then left to soak at room temperature for a period of two hours, with gentle stirring every 30 min to ensure thorough absorption of the nutrient solution. Subsequently, the absorbed agricultural substrate was drained until no free moisture remains on the surface. The substance was transferred into tissue culture flasks and then sterilised via autoclaving at 126 °C for 2 h. This process was intended to eliminate any potential spores or pathogenic microorganisms present in the substrate. Following the attainment of room temperature by the flasks, 5–8 g of mycelium master culture was inoculated within a sterile bench. Subsequently, we transferred the flasks to an incubator that maintained a constant temperature and humidity level of 25 °C and 65% relative humidity, respectively [33]. The culture period lasted 7–10 days, at the end of which the substrate surface was uniformly covered by mycelium and a dense mycelium network had been formed.

It was recommended that the polyester–cotton yarn substrate be pre-treated at the end of the primary culture. The yarn substrate was weighed according to the experimental design ratio, and then soaked in a mixed aqueous solution containing 1% sugar and 1% gypsum powder for a period of 2 h. Following complete absorption of the solution by the yarn, the excess liquid was drained and the material was subjected to high-temperature, high-pressure sterilisation at 126 °C for a duration of two hours. Following a period of cooling to room temperature, the yarn was transferred under sterile conditions into tissue culture flasks containing mycelium-covered crop substrate. Sterile glass rods were utilised to ensure a thorough mixing of the yarn with the crop substrate, thereby facilitating uniform integration. In order to enhance the mixture’s flowability and 3D printability, it was recommended that a xanthan gum and carrageenan solution be added. The mixture was stirred continuously until it formed a uniform, extrusion-ready paste. Subsequently, the material was printed using 3D printing equipment to preset dimensions. The printed secondary culture material was transferred to an incubator that was able to maintain a constant temperature and humidity level of 25 °C and 65% relative humidity (RH). The incubation period was 7–10 days, during which time the mycelium growth was observed at regular intervals to ensure complete penetration and encapsulation of both the yarn and the crop substrate. Following secondary culture, the material was desiccated to yield the mycelium composite.

### 2.3. Research on the Ratio of Polyester–Cotton Matrix to Crop Matrix

As mycelial growth requires abundant carbon and nitrogen sources for nourishment, a certain quantity of agricultural substrate was co-cultivated with the polyester–cotton matrix to produce mycelial composite materials. Prior to the sample preparation, the ratio of yarn to agricultural substrate was investigated. Samples exhibiting optimal growth in both polyester and cotton mycelial composites were deemed the ideal ratio for subsequent experiments. This experiment selected 100% polyester and 100% cotton as textile substrates, establishing five groups with varying yarn-to-crop substrate ratios. Each group utilised 100 g of substrate, as detailed in Table 2.

The experimental samples were printed in the form of 90 mm diameter circles with a thickness of 10 mm. The samples were cultivated for a period of seven days under conditions of 25 °C temperature and 65% relative humidity. The final growth patterns were imported into ImageJ (version 2.3.0, Fiji distribution) image processing software for greyscale image processing, followed by a calculation of the mycelium coverage to determine the optimal ratio of polyester–cotton substrate to agricultural crop substrate. Each ratio sample was subjected to three replicate experiments, and the mean mycelium coverage rate was calculated, as demonstrated in Table 3.

As shown in Table 3, the white areas indicate regions colonised by mycelium, while black areas represent regions not covered by mycelium. Calculating the proportion of white areas reveals that under identical cultivation conditions and duration, both cotton yarn and polyester yarn in Experimental Group B exhibited dense mycelial distribution, achieving mycelial coverage rates of 84.60% and 77.04%, respectively. In all other experimental groups, one type of yarn exhibited poor growth. Consequently, it was determined that the optimal mycelial growth ratio between yarn and agricultural substrate is 1:3. This ratio will be selected for subsequent batch experiments.

### 2.4. Experimental Sample Preparation

**Figure 2:**
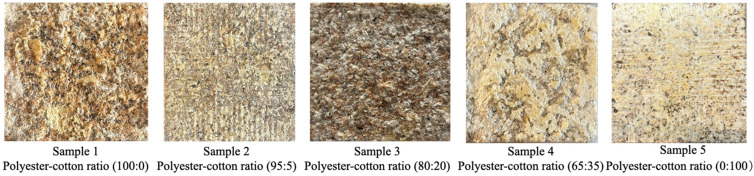
Experimental sample picture.

Common polyester–cotton blend textiles typically feature polyester–cotton ratios of 95:5, 80:20, and 65:35. Consequently, this study utilised five experimental samples with ratios of 100:0, 95:5, 80:20, 65:35, and 0:100, respectively. Samples were numbered from 1 to 5 based on ascending polyester content. Each sample measured 150 mm square with a thickness of 20 mm. Samples were numbered from highest to lowest polyester content, from Sample 1 to Sample 5. Each sample measured 150 mm square with a thickness of 20 mm and was conditioned for seven days at 25 °C and 65% relative humidity. Figure 2 displays images of the experimental samples.

### 2.5. Experimental Test

#### 2.5.1. Hyphal Coverage Rate

During growth, the mycelium envelops the culture medium, forming a dense network structure. The extent of mycelial coverage determines the quality of material growth [34,35], which in turn influences the material’s various properties. Therefore, the experiment utilised ImageJ image processing software to perform greyscale processing on sample images for calculating mycelial coverage. The software automatically calculates the pixel area of black mycelial regions relative to the total pixel area of the entire valid analysis region based on a preset threshold. Finally, the ratio of these two values represents the mycelium coverage rate, calculated as follows: the mycelium coverage rate is equal to the pixel area of the mycelium region, divided by the total pixel area of the effective analysis region, multiplied by 100%. The experiment was conducted with triplicate replicates for each sample. The mean mycelium coverage rate was calculated and expressed as a percentage.

#### 2.5.2. Mechanical Properties Testing

(1) Compression Strength Testing

Mycelium composites are frequently regarded as environmentally friendly alternatives to conventional foam plastics [36,37]. Consequently, this experiment assessed the compressive strength of polyester–cotton matrix mycelium composites by reference to the ISO 604:2002 standard [38] for testing the compressive properties of plastics. Testing was conducted using an SS-8600-1KN universal testing machine at a test rate of 1.00 mm/min. Utilising the formulae for compressive stress (σ) and strain (ε), the force–displacement curve was converted into a stress–strain curve to reveal the material’s failure mode. The compressive strength value was determined by calculating the ratio of the force applied during testing to the specimen’s cross-sectional area, in accordance with Formula (1). Five replicates were performed for each sample, with results expressed in MPa.

(1) σ=F/A

where F represents the measured force (N); A signifies the cross-sectional area of the specimen (mm^2^).

(2) Flexural Strength Testing

Mycelium composites, as a novel bio-based composite material, exhibit mechanical properties comparable to plastics [10,39]. Consequently, testing was conducted in accordance with the ISO 178:2019 standard [40] for plastic flexural properties. An SS-8600-1KN universal testing machine was employed, with displacement time controlled at 2.00 mm/min and a span of (91 ± 0.1) mm. By plotting the stress–strain curve derived from measuring the applied force and deformation during testing, the maximum flexural strength was determined to evaluate the material’s performance under bending loads. The flexural strength calculation formula is shown in Equation (2). Each sample underwent five replicate tests, with results expressed in MPa.

(2) $$\sigma f=3Fl/2{\mathrm{bh}}^{2}$$

where F represents the applied force (N); l indicates the span length (mm); b denotes the specimen width (mm); and h denotes the specimen thickness (mm).

(3) Impact Performance Testing

Mycelium composite materials, owing to their lightweight nature and excellent mechanical properties, find extensive applications in packaging, home furnishings, and other sectors, serving as novel eco-friendly household products [41,42]. Considering the intended application domains of this material, the impact resistance test standard ISO 7765-1:1988 [43]. Plastics—Films and Sheeting—Determination of impact resistance by the free-falling dart method was selected for reference, in conjunction with ISO 4211-4 [44]. Furniture—Tests for Surfaces—Part 4: Assessment of Resistance to Impact Standard to evaluate the material’s impact performance in domestic applications. Impact performance testing of the mycelium composite material was conducted using a BMC-AB drop impact tester (Labthink Instruments Co., Ltd., Jinan, China). Solid balls weighing 70 g, 90 g, 110 g, and 130 g were used to impact the material surface. Impact performance was judged by assessing cracks and damage marks. Five evaluation grades were established to determine the extent of impact damage, as shown in Table 4. Each sample underwent five replicates in this experiment, with results expressed in points.

#### 2.5.3. Determination of Water Vapour Transmittance

Mycelium composites find applications in packaging, home furnishings, and other sectors, where their moisture resistance impacts product longevity and performance. To assess the moisture barrier properties of polyester–cotton matrix mycelium composites, testing was conducted in accordance with the GB/T 1037-2021 Standard [45]. Test Method for Water Vapour Permeability of Plastic Films and Sheets. This standard offers high adaptability, enabling quantification of water vapour permeation rates and direct evaluation of material moisture resistance. Testing was conducted using a WVTR-920-3s water vapour transmission rate tester at 25 °C and 90% relative humidity. The permeation rate was calculated from the sample’s permeated area and test duration, as per Formula (3). Each sample underwent five replicate tests, with results expressed in g/m^2^·24 h.

(3) WVT=24·∆m/A·t

where t represents the time interval between two mass increment stabilisations (h); ∆m denotes the mass increment within time interval t (g); A denotes the area of the specimen permeating water vapour (m^2^).

#### 2.5.4. Determination of Soil Degradation Weight Loss Rate

Mycelia possess the capacity to degrade complex constituents within substrates. To investigate the biodegradability of polyester–cotton substrate mycelium composite materials, soil degradation testing was conducted in accordance with GB/T 19275-2003:Evaluation of the potential biodegradability and disintegration of plastic materials by the action of the specific microorganisms [46]. This standard simulates microbial activity in real soil environments by placing materials within nylon nets. The degradation rate is calculated by measuring mass loss before and after burial in soil, thereby assessing the natural degradation behaviour of the polyester–cotton substrate mycelium composite material. The test employed soil with 100% water content, with a burial period of 28 days. The mass-loss rate was calculated using the formula shown in Equation (4). Each sample underwent five replicates in this experiment, with results expressed as a percentage.

(4) $$m=\frac{m_{1}-m_{2}}{m_{1}}\times 100\%$$

where m_1_ represents the sample mass before degradation (g); m_2_ denotes the sample mass after degradation (g).

### 2.6. Statistics and Analysis

The present study employed the analysis of variance (ANOVA) to analyse whether the polyester–cotton ratio significantly influenced the performance test results of mycelium composite materials. The process comprised four distinct steps. Firstly, model assumption verification was conducted in order to ensure data compliance with the prerequisites for variance analysis. Subsequently, single-factor ANOVA was performed for significance testing to assess the overall impact of the polyester–cotton ratio. When significant results were obtained, post hoc comparison methods were further applied to identify the specific sources of variation. Finally, effect sizes were calculated in order to quantitatively assess the practical significance of the influencing factors.

This study employed quantitative statistical analysis methods, with a significance level set at *p* < 0.05. Data normality was verified via the Shapiro–Wilk test (*p* > 0.05), and Levene’s test confirmed the homogeneity of variance (*p* > 0.05). An appropriate ANOVA method was selected based on the experimental design. Where ANOVA results indicated significant intergroup differences (*p* < 0.05), post hoc tests were conducted for multiple comparisons. ANOVA analysis quantified the effect size of the polyester–cotton ratio on material properties, thereby determining the statistical significance of performance variations across different polyester–cotton ratios.

## 3. Results and Discussion

### 3.1. Results

#### 3.1.1. Mycelium Coverage Rate

Mycelial growth is found to be significantly influenced by the composition of the fibre. The greyscale images and mycelial coverage of experimental samples are shown in Table 5. Samples 4 and 5 demonstrated optimal mycelium proliferation in composite materials, with mycelium almost completely enveloping the culture substrate. The results of three replicate experiments yielded mycelial coverage percentages of 83.88% and 86.32%, respectively. Consequently, a higher cotton fibre content has been demonstrated to correlate with enhanced mycelium growth. This phenomenon is attributed to the inherent properties of cotton yarn, which are characterised by a high content of lignin and cellulose, in conjunction with exceptional heat and moisture retention capabilities. Amongst agricultural substrates exhibiting identical content ratios, mycelium composite materials containing elevated levels of cotton demonstrated superior growth characteristics. Mycelium in polyester-rich composites also bound loose fibres together, but achieved lower coverage.

#### 3.1.2. Mechanical Properties

(1) Compression Strength

**Figure 3:**
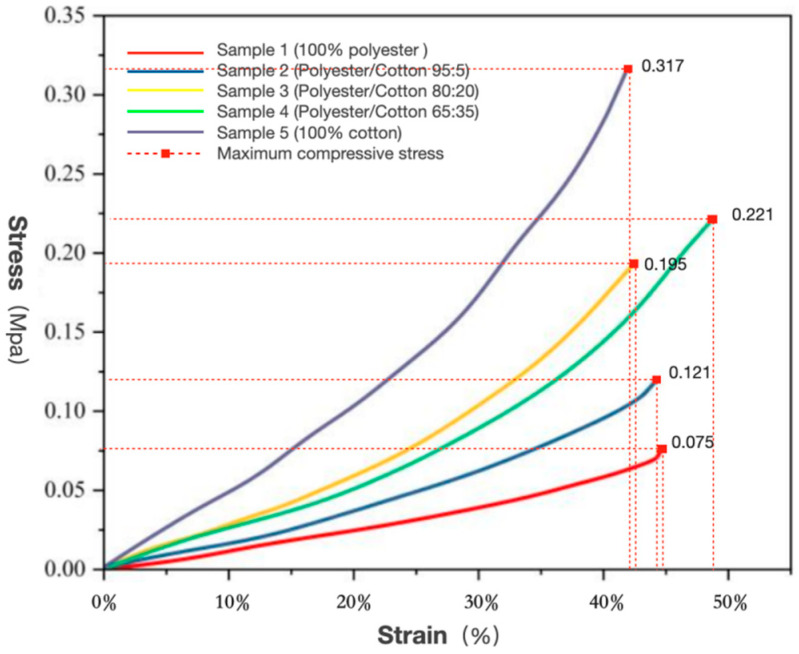
Compression stress–strain curves of mycelium composite materials with five kinds of polyester–cotton matrix ratios.

As demonstrated in Figure 3, the compression stress–strain curves for five mycelium composites with varying polyester–cotton ratios are presented, visually illustrating the response patterns of strain and stress during compression. In the initial phase of the study, the mycelium composites that were tested with different polyester–cotton matrices exhibited similar compression behaviour. However, as the strain increased, performance differences became more apparent. Among the samples examined, the one composed of 100% polyester exhibited the most gradual increase in compressive stress. As the strain approached 50%, the stress registered at approximately 0.075 MPa, indicating its relatively weak ability to withstand compressive stress. An increase in cotton content from 0% to 35% was observed to result in a rise in compressive stress from 0.075 MPa to 0.317 MPa. This finding indicates a substantial enhancement in the material’s compressive performance. This finding suggests that the mechanical properties of mycelium composites are significantly influenced by the substrate, with the ratio of cotton to polyester playing a crucial role in material performance. Cotton fibres offer superior moisture absorption and breathability, and their rich lignocellulose content provides ample nutrients for mycelial growth [47,48], facilitating the formation of high-density mycelial networks. Furthermore, the combination of cotton’s flexibility and its ability to form strong interfacial bonds with mycelium has been shown to enhance the overall performance of the composite. This is due to the fact that cotton’s inherent properties, when combined with the high strength and modulus of polyester, result in a composite material that exhibits superior resistance to compressive loads.

However, when the cotton content was increased from 35% to 100%, the compressive stress decreased to 0.221 MPa, indicating a decline in the composite’s compressive performance. This phenomenon can be attributed to the fact that, while polyester is characterised by certain limitations in terms of structural toughness, its high strength and modulus provide essential rigid support to the composite. In contrast, cotton fibres exhibit comparatively low tensile strength; when their proportion exceeds a certain threshold, they are unable to provide effective support that is commensurate with that of polyester, thereby reducing the material’s capacity to withstand compressive stress. Consequently, an increase in the cotton content results in enhanced compressive strength of the material; however, it should be noted that higher is not necessarily better in this regard. In order to achieve optimal compressive performance in the composite material, it is essential to employ a balanced blend, comprising an appropriate amount of polyester fibre.

(2) Flexural Strength

**Figure 4:**
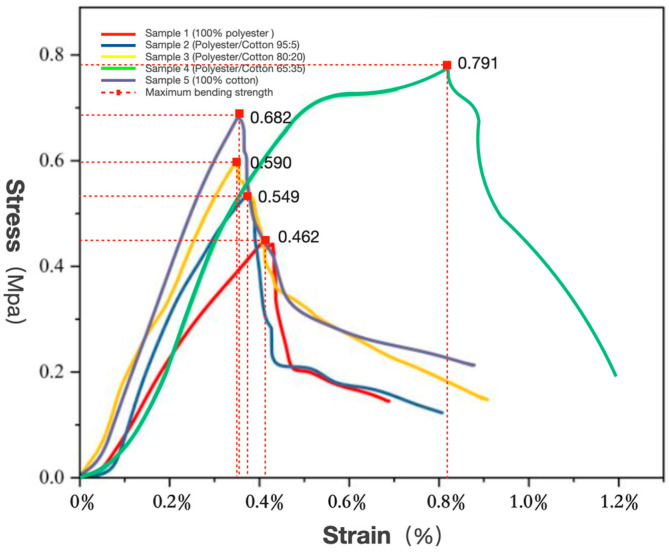
Bending stress–strain curves of mycelium composite materials with five kinds of polyester–cotton matrix ratios.

To clarify the influence of the polyester–cotton matrix ratio on bending properties, an analysis of their bending stress–strain behaviour was conducted, as illustrated in Figure 4. During the initial strain phase, the curves of all five samples exhibited a high degree of overlap. As strain increased, differences gradually became apparent; upon reaching approximately 0.4% strain, samples 1, 2, 3, and 5 all attained their yield points, at which point the specimens sustained bending failure. The maximum bending strengths recorded were 0.462 MPa, 0.549 MPa, 0.590 MPa, and 0.682 MPa, respectively; at a strain of 0.82%, Sample 4 reached its yield point with a flexural strength of 0.791 MPa. Sample 4 exhibited a steeper curve slope, indicating greater flexural strength and enhanced bending resistance for the same deformation.

The polyester–cotton ratio influences the material’s flexural strength. As polyester content decreases, flexural strength exhibits an upward trend, suggesting a correlation between compressive strength and flexural strength. Concurrently, moderate polyester content enhances the toughness of mycelium composite materials [49,50]. However, materials composed solely of polyester exhibiting low flexural strength, reaffirming that pure polyester matrices are unsuitable as growth substrates for mycelium composites. Increased cotton content correlates with improved flexural performance, with Sample 4 demonstrating superior properties to Sample 5. This may stem from the absence of polyester reinforcement in pure cotton fibres, allowing their inherent flexibility to dominate.

(3) Impact Performance

The test images and scores for polyester–cotton matrix mycelium composite materials at different grammages, both prior to and following impact, are displayed in Table 6. In conditions of force below 70 g, the five materials exhibited negligible changes. At a force of 90 g, Samples 1 and 2 exhibited a decline in performance, accompanied by the emergence of minor cracks due to impact stress. In contrast, Samples 3, 4, and 5 demonstrated no perceptible change. At an applied force of 110 g, Samples 1 and 2 fractured completely, resulting in material failure, while Samples 3, 4, and 5 exhibited varying degrees of cracking. At a force of 130 g, Samples 1, 2, and 3 all failed, while Samples 4 and 5 exhibited cracks but did not fracture.

It is evident from the total scores that the impact rating exhibited an increase in proportion with rising cotton content. This finding suggests that the proportion of cotton in the polyester–cotton matrix exerts a significant influence on the impact resistance of the mycelium composite material. It has been demonstrated that an increased cotton fibre content results in enhanced material impact resistance. It has been demonstrated that when the cotton content exceeds 35%, there is a 25% improvement in impact performance. This phenomenon can be attributed to the inherent properties of cotton fibres, which are characterised by their high lignocellulose content. These fibres have been observed to facilitate enhanced mycelium integration with yarns, thereby promoting the formation of dense mycelium networks. These networks, in turn, have been shown to strengthen interfacial bonding, contributing to the overall integrity and strength of the yarn. In contrast, polyester fibres have been found to be deficient in nutrients that are conducive to mycelium growth. This deficiency can lead to suboptimal interface compatibility and diminished impact resistance.

In summary, the incorporation of cotton fibres during the cultivation of mycelium composites enhances the material’s impact resistance. Cotton fibres have been demonstrated to enhance mycelium growth, thereby optimising material flexibility and compensating for the limitations of pure polyester materials. The optimal effect is achieved when the cotton content exceeds 35%, providing a basis for the optimisation of substrate composition.

#### 3.1.3. Water Vapour Transmittance

**Figure 5:**
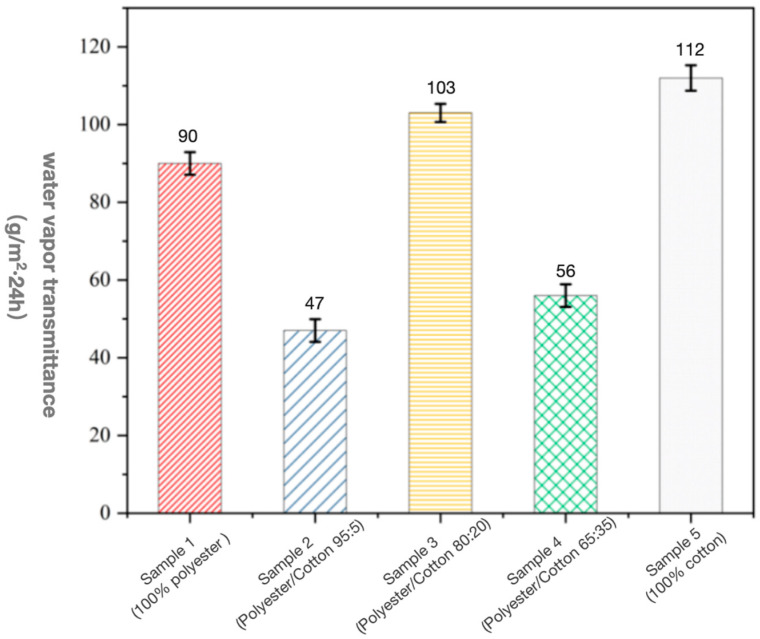
Water vapour transmission rate of mycelium composite materials with different polyester–cotton ratios.

As shown in Figure 5, the results of water vapour transmission rate tests for mycelium composites with varying polyester–cotton ratios are presented. Lower values indicate superior moisture resistance, with Samples 2 and 4 demonstrating the best performance at 47 g/m^2^·24 h and 56 g/m^2^·24 h, respectively, while samples 1, 3, and 5 recorded vapour permeation values of 90 g/m^2^·24 h, 103 g/m^2^·24 h, and 112 g/m^2^·24 h, respectively. This indicates that pure cotton matrix materials exhibit the highest water vapour permeability among all fibres, while polyester-matrix materials demonstrate relatively lower permeability. Consequently, composite materials with higher polyester content in the mycelium matrix demonstrate superior moisture resistance [51,52]. However, the vapour permeability values across the five samples did not exhibit a straightforward linear relationship; lower cotton content did not necessarily correlate with superior moisture resistance. Notably, Sample 1 (pure polyester) demonstrated inferior moisture resistance compared to Sample 2 (5% cotton blend). This indicates that blending a small proportion of cotton fibres with high polyester content optimises moisture resistance. This may occur because cotton fibres fill the voids in polyester fibres, thereby blocking water vapour transmission. As the cotton content increases, the water vapour transmission rate exhibits a pattern of initial increase, followed by a decrease, and then another increase. This reflects that the polyester–cotton blend ratio must be appropriately matched to achieve a balanced moisture-resistance performance.

#### 3.1.4. Soil Degradation

**Figure 6:**
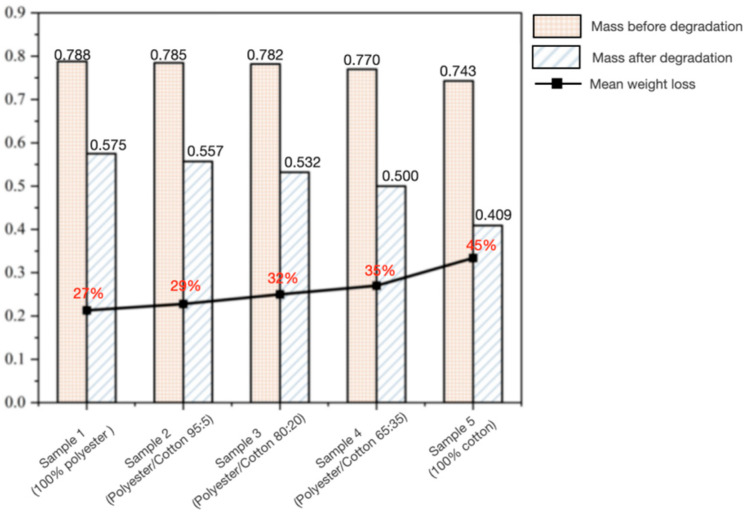
Test results on the degradability of mycelium composites with different polyester–cotton ratios.

As shown in Figure 6, the results of soil degradation weight loss tests for mycelium composites with varying polyester–cotton ratios are presented. Pre-degradation mass exhibited slight variations depending on cotton content proportion. Post-degradation mass demonstrated a trend where higher cotton content corresponded to lower values, whilst the average mass loss progressively increased with rising cotton proportion. Sample 5 exhibited an average weight loss of 45%, significantly exceeding that of Sample 1 (27%). This indicates that a higher cotton proportion within the mycelium composite enhances its biodegradability. This effect arises because cotton fibres are primarily composed of cellulose, a natural polymeric polysaccharide [53] and one of nature’s most abundant organic compounds. Cellulose readily decomposes into glucose through action by cellulase enzymes secreted by soil microorganisms, subsequently becoming utilised by these microbes. When mycelium binds with the culture substrate, it releases chitinase, which degrades and transforms the substrate [54]. Polyester, primarily composed of polyethylene terephthalate (PET), is chemically synthesised from petroleum-based feedstocks. It exhibits high hydrophobicity and resistance to biodegradation. Soil microorganisms lack enzymes capable of effectively decomposing polyester, resulting in extremely slow degradation rates in natural environments—sometimes requiring decades for complete breakdown [55,56]. However, experimental evidence indicates that polyester-matrix mycelium composite materials exhibit certain biodegradable properties. Cotton fibres demonstrate superior degradability compared to polyester fibres, and even minimal incorporation of cotton fibres significantly enhances the overall degradability of the material. This provides quantitative evidence for optimising the degradation characteristics of such materials.

It is evident from the experimental findings on the aforementioned properties that the optimal loading level for the polyester–cotton matrix mycelium composite material is Sample 4, which exhibits a polyester–cotton ratio of 65:35. With regard to mycelium growth and mechanical properties, this ratio achieves high mycelium coverage, approaching the level of the high-cotton group. This enables the formation of a dense mycelium network that provides a reliable structural foundation for the material’s mechanical properties. With regard to compressive performance, the material displays a compressive stress that significantly exceeds that of both the pure polyester and pure cotton groups. This is indicative of a synergistic complementarity between the flexibility of cotton fibres and the rigidity of polyester fibres. The bending performance of the material is optimal, with bending strength exceeding that of all other groups, and a later yield stress demonstrating superior bending resistance. Furthermore, the impact resistance of the samples was found to be of a superior calibre, exhibiting only fissures without fracture under high-load impacts. Its rating was second only to the high-cotton group. Furthermore, it was demonstrated that when cotton content exceeded 35%, the material’s impact resistance significantly improved, achieving a balance between excellent toughness and fracture resistance.

With regard to moisture resistance and biodegradability, while this blend does not demonstrate the highest level of resistance, its water vapour transmission rate remains low enough to meet the fundamental moisture protection requirements. Moreover, it circumvents the poor interfacial compatibility and weak mechanical properties that are characteristic of the pure polyester group. With regard to the question of biodegradability, while the degradation efficiency of the material is lower than that of pure cotton, it is significantly superior to that of pure polyester. This represents an effective balance between material mechanical properties and environmental friendliness.

#### 3.1.5. ANOVA Analysis

To investigate the extent to which the polyester–cotton ratio influences material properties, statistical analysis was conducted using ANOVA. Data from five replicate experiments per sample served as the statistical basis through following Shapiro–Wilk normality testing and a homogeneity of variance test, followed by ANOVA and Welch ANOVA analysis. This yielded the effect size ηp^2^ for the influence of the polyester–cotton matrix ratio on individual properties, thereby determining the statistical significance of differences under various ratios, as shown in Table 7.

As shown in Table 7, in the analysis of compression performance, most samples satisfied normal distribution except for Sample 4 (*p* = 0.033). The test for homogeneity of variances (*p* = 0.00) selected Welch ANOVA analysis under non-homogeneity. The polyester–cotton ratio exerted an extremely significant influence (effect size ηp^2^ = 0.999804). Post hoc tests revealed extremely significant differences between all groups, except for the near-significant margin observed between Samples 3 and 4 (*p* = 0.047). Regarding bending performance, all samples conformed to normal distribution with homogeneity of variance. Both ANOVA and Welch ANOVA validated significant inter-group differences (effect size ηp^2^ = 0.948), indicating that polyester–cotton ratio explained 94.78% of bending performance variance. Post hoc tests revealed significant differences between all groups except Sample 4 and Sample 5. Impact performance testing showed most samples (except Sample 3) satisfied normality and homogeneity of variance. Both ANOVA and Welch ANOVA revealed significant intergroup differences, with an effect size ηp^2^ = 0.841. This indicates that the polyester–cotton ratio explains 84.1% of impact performance variation. In moisture resistance testing, all samples exhibited normal distribution and homogeneity of variance. Both ANOVA and Welch ANOVA indicated significant inter-group differences, with an effect size of ηp^2^ = 0.990, demonstrating that the polyester–cotton ratio explains 99% of the variation in moisture-resistance performance. For the biodegradability dimension, samples satisfied normal distribution and homogeneity of variance. Both ANOVA and Welch ANOVA revealed extremely significant inter-group differences, with an effect size of ηp^2^ = 0.992. This indicates that the polyester–cotton ratio explains 99.1% of the variation in biodegradability performance. Through multidimensional testing, the role of the polyester–cotton ratio across different performance dimensions is clearly demonstrated, providing foundational data support for subsequent performance regulation.

Effect size comparisons indicate that compression performance (1.000), moisture resistance (0.990), and biodegradability (0.992) all exceed 99% explanatory power, while bending performance (0.948) and impact resistance (0.841) show progressively lower explanatory capabilities. Consequently, it is determined that the polyester–cotton ratio exerts the most significant influence on compression performance. Simultaneously, it exerts a highly significant effect on moisture resistance and biodegradability while exhibiting a gradient difference in its impact on bending and impact performance. Overall, this validates that the polyester–cotton ratio is the core factor in regulating the performance of textile matrix mycelium composite materials.

### 3.2. Discussion

The present study involved the preparation of multiple mycelium composite samples, characterised by varying polyester–cotton ratios. The performance data for these samples are presented in Table 8. The findings suggest that this material achieves an optimal balance between environmental sustainability and mechanical utility, exhibiting properties comparable to various traditional materials. This finding indicates its potential for future application as a substitute material, as illustrated in Table 9.

This material’s most significant advantage lies in its exceptional biodegradability. In soil, it degrades by 27% to 45% in just 28 days, enabling it to break down quickly in natural environments. This is in stark contrast to traditional expanded polystyrene and polyurethane materials, which take decades or longer to degrade. In the fields of cushioning packaging and decorative materials, this material offers unparalleled environmental benefits. In terms of mechanical properties, while its compressive and flexural strengths are lower than those of structural engineered wood panels, its performance matches and even exceeds that of general-purpose expanded polystyrene. Impact resistance testing confirms that this material has the mechanical properties necessary to serve as an alternative to conventional plastic foam for cushioning packaging. However, this material also has some drawbacks. For example, it has a high water vapour permeability rate, indicating poor moisture resistance, which limits its use in areas such as electronics and dry food packaging. Nevertheless, this characteristic also makes it suitable for applications requiring high breathability. Future research could enhance moisture resistance further through post-treatment techniques such as hydrophobic surface coatings. Overall, this study successfully developed an eco-friendly material offering both practical performance and rapid biodegradability by optimising the polyester–cotton blend ratio. This provides a new technical solution for textile waste reuse and green material development.

## 4. Design Applications

The preparation of mycelium composites using polyester–cotton substrates offers an innovative approach to waste-textile processing. Experimental findings indicate that mycelium composites with a polyester–cotton substrate ratio of 65:35 exhibit superior performance advantages. This ratio effectively balances the relationship between polyester–cotton proportions and material properties, providing a practical solution for product applications.

As a growth-based biomaterial, mycelium composites offer novel visual experiences during their development. This paper therefore employs the material to develop experiential products, inviting consumers to participate in the manufacturing process and appreciate the unique sensory engagement it provides. Simultaneously, these products utilise waste textile materials as their matrix. This approach not only provides a new pathway for recycling textile waste but also promotes eco-friendly materials through participatory experiences. This subtly instils sustainable values, achieving mutual empowerment between environmental benefits and user experience. The process begins by defining the target user group and design requirements, followed by identifying the product’s application scenarios before proceeding with design implementation. The satisfaction levels of the experiential product design solutions were subsequently evaluated using Likert scales and the mean method.

### 4.1. User Demand Survey

This project targets the 18–26 age group as its primary consumer demographic. This cohort demonstrates greater receptiveness to novelty, pursues niche cultures, and places heightened emphasis on immersive consumption experiences. They favour highly interactive design formats, with such experiences not only fulfilling their entertainment needs but also evoking emotional resonance and cultural identification. When purchasing products, this demographic demonstrates a marked preference for environmentally sustainable options and is willing to pay a premium for goods that align with their personal values. Research into this user group reveals that 68.4% prioritise participatory design elements, followed by 64.62% who value the product’s sense of playful enjoyment. Cost-effectiveness and material quality represent underlying preferences.

### 4.2. Product Application Scenarios

In modern society, individuals often find themselves under high pressure and tension in external settings, such as work and commuting. Upon returning home, however, they experience relatively concentrated periods of leisure time in which relaxation prevails. Mycelium composite experiential products require users to dedicate a certain amount of time to complete the cultivation experience and participate in the design process, demanding phased attention throughout the entire journey. Within the domestic setting, users are free from time constraints or social pressures, enabling them to savour the experience process with greater composure. These fragmented moments of leisure are imbued with a restorative quality, fostering a sense of communion with nature. Consequently, the domestic environment has been selected as the primary application scenario for experiential cultural and creative design in this research.

Home décor often embodies the homeowner’s aesthetic expression. While conventional cultural products feature pre-determined aesthetics, experiential products cultivate beauty collaboratively. Taking mycelium composite materials as an example: users observe unique textures formed by spreading mycelium during cultivation, then adjust assembly patterns according to personal taste during assembly. The final product’s beauty transcends mere visual appeal, incorporating the emotional value of the creative process. This experiential process precisely aligns with the personalised decorative demands of domestic settings, elevating products from mere commodities to aesthetic symbols imbued with emotional resonance. They thus establish a warmer, more harmonious dialogue with the furnishings, colour palettes, and stylistic elements within the home environment.

### 4.3. Design Practice

This research develops mycelium-based composite materials utilising recycled polyester–cotton substrates, creating experiential home décor products from a product application perspective. Centred on experiential design and sustainable design principles, three distinct product series have been developed to achieve a serialised design. By enabling experiential engagement with this material, sustainable innovation is realised, bringing living biomaterials into close proximity with daily life. This enhances consumers’ awareness and understanding of eco-conscious design and ecological principles.

#### 4.3.1. Inspiration Source and Transformation of Design Elements

This design draws inspiration from Dalian’s distinctive regional culture for home furnishings, transforming concrete architectural landscapes and urban elements into rounded cartoon-style graphics that better align with the aesthetic preferences of younger demographics. As shown in Table 10, this illustrates the extraction of architectural inspiration and its conversion into geometric forms.

#### 4.3.2. Product Design

**Figure 7:**
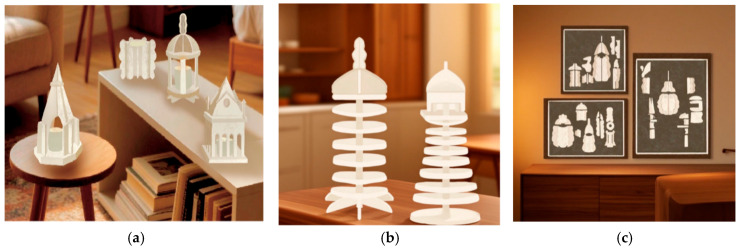
Polyester–cotton base mycelium composite material experiential home products. (**a**) Mycelium mosaic in an aromatherapy box; (**b**) mycelium mosaic ornaments; (**c**) decorative painting of mycelium mosaic.

This experiential homeware collection comprises three distinct series: interlocking flameless fragrance boxes, interlocking decorative artworks, and interlocking ornaments. The plug-in flameless aroma diffuser employs a multi-sensory design approach, prioritising the tactile experience, with visual elements as a secondary focus. By incorporating olfactory stimulation into the diffuser’s design, it delivers an immersive experience across multiple dimensions. From a product form perspective, the design abstracts and transforms the architectural silhouettes of Dalian into three-dimensional motifs, as illustrated in Figure 7a. Two decorative ornaments were designed, drawing inspiration from Dalian’s iconic lighthouse silhouettes. These were reinterpreted through contemporary design language. Employing a modular, detachable structure composed of circular units of varying sizes, interconnected via interlocking mechanisms, they achieve aesthetic home décor functionality, as illustrated in Figure 7b. The three interlocking decorative panels designed here deconstruct Dalian’s regional graphic elements into semi-three-dimensional modular pieces. Patterns emerge through the assembly of multiple unit modules. These designs are broken down into 20–30 mycelium composite modules, each maintaining uniform thickness. Users can freely combine and assemble them to recreate standard patterns or exercise creativity through personalised arrangements, as illustrated in Figure 7c.

#### 4.3.3. Experience Mode

**Figure 8:**
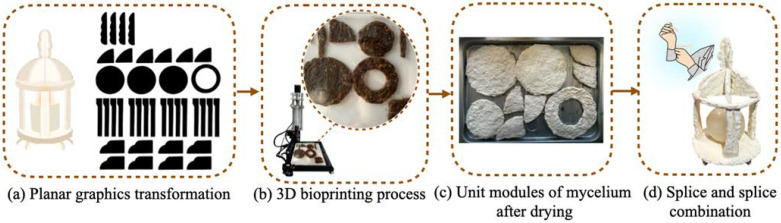
Experience mode of terylene–cotton matrix mycelium composite household products.

The primary experience model for this home product involves the processes of assembly and construction. Printed materials are sold to consumers, who then assemble them into cultural and creative products for use. The process of printing is facilitated by the aforementioned 3D printing technology, which employs a self-assembled 3D printer composed of a typewriter and an automatic injection pump. The experience model is illustrated using an aromatherapy box as an example. The three-dimensional incense box is decomposed into multiple flat geometric shapes. These are imported into CORDRAW2019 software for dimensional editing, as demonstrated in Figure 8a. Subsequently, the flat geometric shapes are entered into the printer’s integrated programme. Following the adjustment of the graphic size, the printing process is initiated concurrently with the simultaneous control of the automatic syringe pump to extrude mycelium slurry. It is evident that due to the prevailing technical and equipment limitations, the execution of printing processes is constrained to flat single-layer printing, as illustrated in Figure 8b. Subsequent to the printing process, the culture tray is placed within a constant temperature and humidity incubator for a period of seven days at a temperature of 25 °C and a relative humidity of 65%. The process of cultivation is deemed to be complete once mycelium has been observed to fully cover the surface. The material is then subjected to oven-drying at a temperature of 110 °C for a duration of two hours. Thereafter, it is left to acclimatise under natural conditions to facilitate the complete evaporation of internal moisture, as illustrated in Figure 8c. The material is then assembled by users according to the instructions for joining, with the resultant products then being used for home decor, as illustrated in Figure 8d.

### 4.4. Experience Programme and Evaluation

To measure consumer satisfaction with experiential product design, the outcomes of design proposals must be evaluated. The rationality of design requires assessment through composite metrics, as single evaluation criteria cannot comprehensively reflect overall design quality. Therefore, multi-dimensional evaluation metrics—covering product sustainability, experiential value, and aesthetic appeal—provide a decision-making basis for experiential product design.

Currently, no unified evaluation standards exist for experiential products. This study employs methods including focus group discussions, user surveys, and expert consultations. Based on the various dimensions of experiential cultural and creative products, evaluation criteria for design proposals are formulated, as shown in Table 11. A total of nine evaluation standards are established.

A five-point Likert scale questionnaire was employed to evaluate the three products developed during this design practice. The ratings ‘1–5’ corresponded to the assessment criteria ‘very dissatisfied’, ‘dissatisfied’, ‘neutral’, “satisfied”, and ‘very satisfied’, respectively, indicating respondents’ level of satisfaction with the design proposals. A total of 55 valid questionnaires were collected from participants comprising consumers, undergraduate design students, postgraduate design students, and specialist teaching staff. Online SPSSAU analysis yielded a Cronbach’s alpha coefficient of 0.85, indicating good scale reliability, while the KMO value of 0.83 demonstrated excellent suitability of the research data for information extraction.

The mean method was employed to statistically analyse satisfaction across nine experiential cultural and creative design evaluation indicators. The composite scores for the three schemes were 4.18, 3.76, and 4.02, respectively, as shown in Table 12. The interlocking flameless incense box design achieved the highest satisfaction rating, demonstrating outstanding performance in material efficiency and reuse, thereby embodying excellent sustainable design characteristics. It also received high ratings for aesthetic appeal and emotional resonance, indicating a successful balance between environmental attributes and user experience. In contrast, the decorative ornament scored lowest overall, primarily constrained by its lack of visual appeal and interactive engagement, reflecting the adaptive challenges of traditional form-based design when applied to novel biomaterials. All three product designs demonstrated room for improvement in material lifecycle management and industrialisation potential. Overall, the experiential home, cultural, and creative product design proposals proved largely satisfactory, achieving the anticipated design objectives.

## 5. Conclusions

The present study prepared mycelium-based composite materials using a polyester–cotton matrix as the substrate, thus offering an innovative approach for the treatment of textile waste. In order to ascertain the impact of the polyester–cotton ratio on material properties, five composite materials were prepared, each with a different polyester–cotton ratio. In addition to this, mechanical property, moisture resistance, and biodegradability testing was conducted, alongside analysis of the results using ANOVA. The results of this study confirmed that the polyester–cotton ratio has a significant impact on material properties. The most significant effect was observed on compressive strength, followed by moisture resistance and biodegradability. Specifically, an elevated cotton fibre content enhances compressive strength but reduces moisture resistance; conversely, an increased polyester fibre content improves moisture resistance but diminishes mechanical properties and biodegradability. In general, the composite material with a 65:35 polyester–cotton ratio demonstrated an optimal performance, attaining a compressive strength of 0.221 MPa and a flexural strength of 0.791 MPa. Furthermore, the material demonstrated excellent impact resistance, moisture resistance, and biodegradability, rendering it suitable for utilisation in home decor products. The experiential home products that were developed based on the material’s growth characteristics also provide direction for its application in design fields.

The present study focused exclusively on polyester–cotton matrices with limited mixed fibre types. It is recommended that future research endeavours extend the current material preparation experiments to encompass a range of fibre types and precise ratios. This expansion will facilitate the optimisation of the performance of textile matrix–mycelium composites. Further refinement of material application performance testing is required in order to identify additional potential for applications. This method of preparation could be applied to actual textile waste treatment, in order to validate its feasibility in recycling discarded textiles. Concurrently, the integration of diverse design philosophies to explore varied design approaches could advance the application of textile waste resources in sustainable eco-materials, thereby supporting sustainable design development within the apparel sector.

## Figures and Tables

**Table 1:** Experimental equipment.

Type of Equipment	Name of Device	Pictures of Equipment	Model Number	Equipment Manufacturer
Crushing equipment	Grinding machine	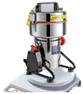	AF-03S	Aoli Trodutional Chinese Medicine Machinery Co. Ltd., Wenling City, China
Sterilisation equipment	Portable pressure steam sterilisation pot	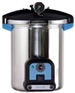	XFS-300	Shangyi Scientific Instrument Co. Ltd., Shanghai, China.
Equipment for inoculation	Bechtop	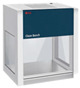	VD650	ShangGuang Instrument Manufacturing Co. Ltd., Shanghai, China.
Printing equipment	Automatic injection device	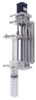	QHZS-001A	Jiaozuo Yanhang Power Technology Co. Ltd., Jiaozuo City, China.
	Writing machine	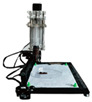	LHB-24	Jinqian Intelligent Technology Co. Ltd., Jinhua City, China.
Equipment for cultivation	Constant temperature and humidity incubator	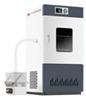	HWS-250	Shangyu Yuanyang instrument business department, Shaoxing, China
Drying equipment	Electrothermal constant temperature air drying oven	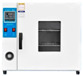	SN-101-0A	Shangyi Scientific Instrument Co. Ltd., Shanghai, China.

**Table 2:** Polyester–cotton yarn to crop matrix ratio data.

Group	Yarn to Crop Substrate Ratio	Yarn and Crop Content (g)	Type of Yarn
Experiment A-Cotton	1:1	50 g of yarn	100% cotton
Experimental A-Polyester		50 g of crop	100% polyester
Experimental B-Cotton	1:3	25 g of yarn	100% cotton
Experimental B-Polyester		75 g of crop	100% polyester
Experimental C-Cotton	1:5	17 g of yarn	100% cotton
Experimental C-Polyester		83 g of crop	100% polyester
Experimental D-Cotton	5:1	83 g yarn	100% cotton
Experimental D-Polyester		17 g of crop	100% polyester
Experimental E-Cotton	3:1	75 g of yarn	100% cotton
Experimental E-Polyester		25 g of crop	100% polyester

**Table 3:** Experimental growth conditions and greyscale images of the proportion of polyester–cotton yarn and crop substrate.

Yarn to Crop Substrate Ratio	Experimental Groups	Final Growth Image	Greyscale Image	Mean Mycelium Coverage
1:1	Experimental group A—100% cotton	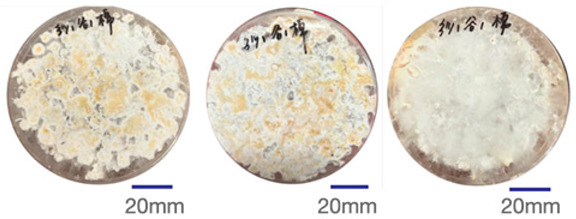	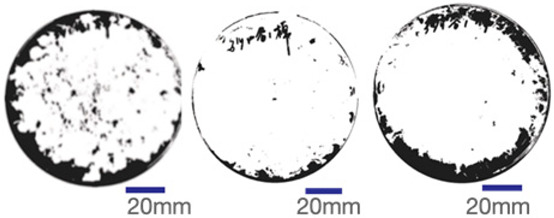	82.87%
	Mycelium coverage rate		77.27% 92.59% 78.75%	
	Experimental group A—100% polyester	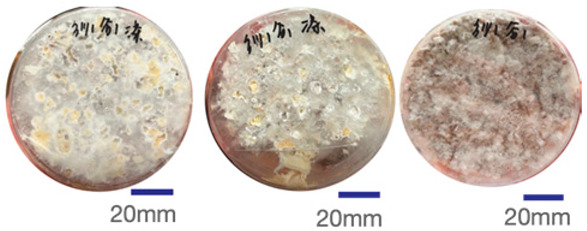	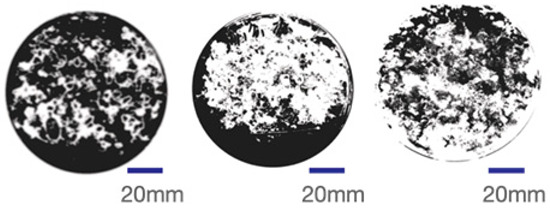	48.67%
	Mycelium coverage rate		32.66% 53.75% 62.6%	
1:3	Experimental group B—100% cotton	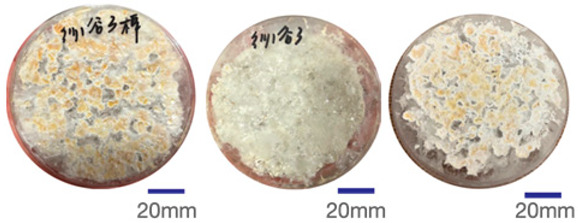	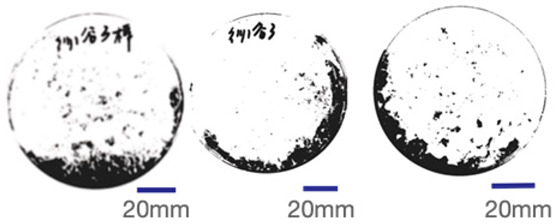	84.60%
	Mycelium coverage rate		84.46% 84.48% 84.85%	
	Experimental group B—100% polyester	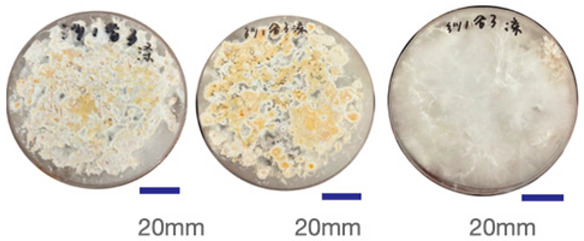	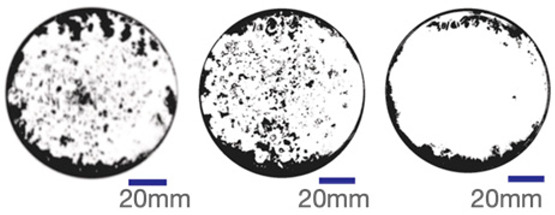	77.04%
	Mycelium coverage rate		73.86% 74.23% 83.02%	
1:5	Experimental group C—100% cotton	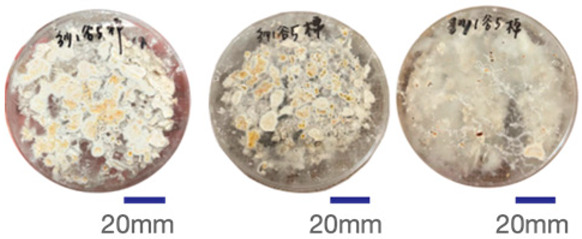	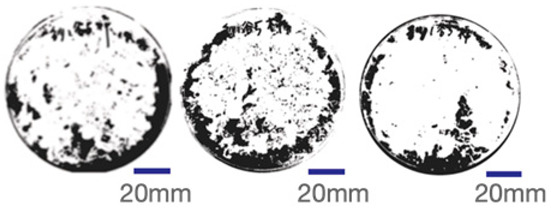	76.25%
	Mycelium coverage rate		76.33% 72.77% 79.66%	
	Experimental group C—100% polyester	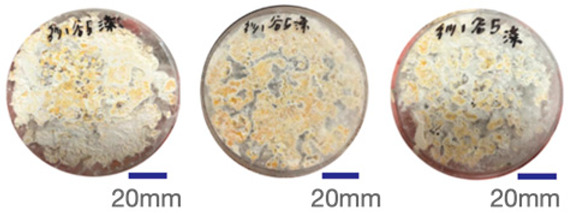	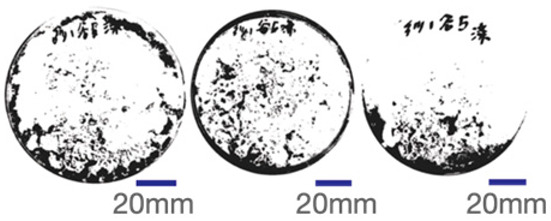	78.64%
	Mycelium coverage rate		78.09% 75.24% 82.60%	
5:1	Experimental group D—100% cotton	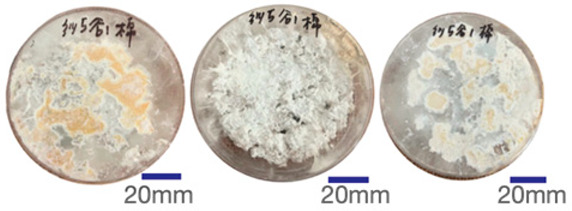	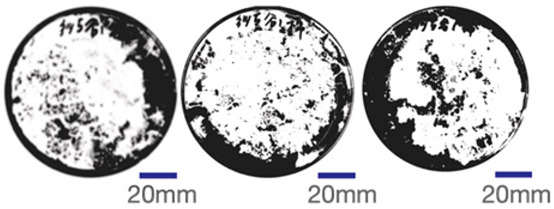	63.03%
	Mycelium coverage rate		62.78% 67.82% 58.49%	
	Experimental group D—100% polyester	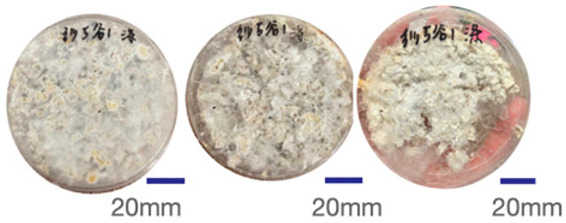	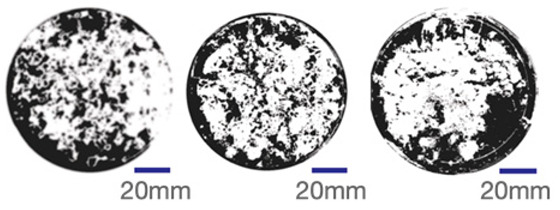	57.46%
	Mycelium coverage rate		57.26% 58.51% 56.60%	
3:1	Experimental group E-100% cotton	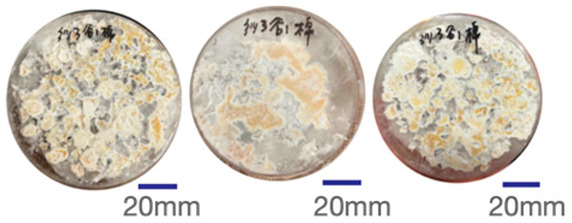	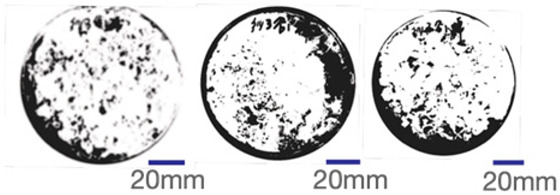	71.93%
	Mycelium coverage rate		78.4% 65.02% 72.37%	
	Experimental group E—100% polyester	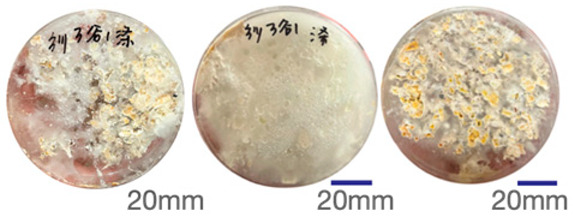	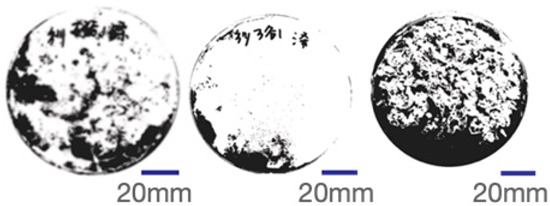	69.60%
	Mycelium coverage rate		69.88% 87.68% 51.23%	

**Table 4:** Impact performance evaluation criteria.

Rating	Description
5	No significant change
4	There are no cracks on the surface, but the impact marks can be seen through the light
3	Slight surface cracks (10–20 mm)
2	Severe surface cracks (21–30 mm)
1	Fracture of material

**Table 5:** The proportion of greyscale images and mycelium.

Repeat Repeat Sample	Sample 1 Polyester–Cotton Ratio (100:0)	Sample 2 Polyester–Cotton Ratio (95:5)	Sample 3 Polyester–Cotton Ratio (80:20)	Sample 4 Polyester–Cotton Ratio (65:35)	Sample 5 Polyester–Cotton Ratio (0:100)
The First Time	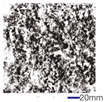	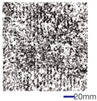	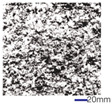	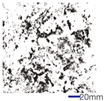	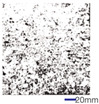
Mycelium Coverage Rate	56.71%	62.99%	63.74%	81.60%	82.73
The Second Time	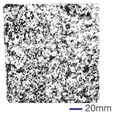	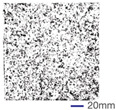	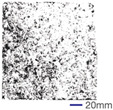	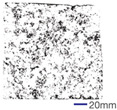	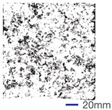
Mycelium Coverage Rate	66.21%	79.11%	80.52%	82.65%	85.22%
The Third Time	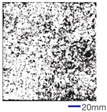	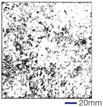	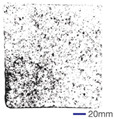	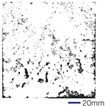	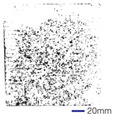
Mycelium Coverage Rate	72.73%	80.30%	85.48%	87.39%	91.00%
Average Value	65.22%	74.13%	76.58%	83.88%	86.32%

**Table 6:** Impact test results of a polyester–cotton matrix mycelium composite material.

Sample Weight	70 g		90 g		110 g		130 g		Total Score
Sample 1 100% Polyester	Before	After	Before	After	Before	After	Before	After	9
	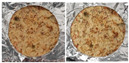		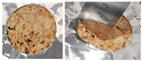		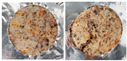		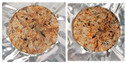		
Score	4		3		1		1		
Sample 2 Polyester/Cotton 95:5	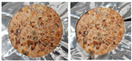		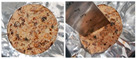		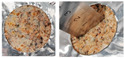		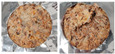		11
Score	5		3		2		1		
Sample 3 Polyester/Cotton 80:20	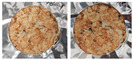		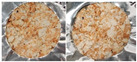		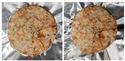		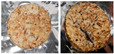		12
Score	4		4		3		1		
Sample 4 Polyester/Cotton 65:35	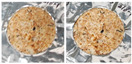		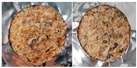		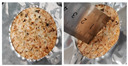		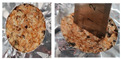		15
Score	5		4		3		3		
Sample 5 100% Cotton	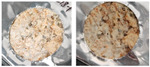		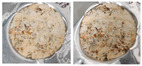		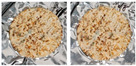		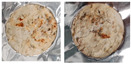		17
Score	5		5		4		3		

**Table 7:** ANOVA analysis results of five performances.

Item of Test	Degree of Freedom	F-Statistic	*p*-Value	ηp^2^
Strength of compression	10.517	10,395.335	4.692797 × 10^−19^	1.000
Strength of bending	10.723	139.913	2.157117 × 10^−9^	0.948
Performance of impact	11.016	39.179	0.00	0.841
Water vapour transmittance	10.517	10,395.335	4.692797 × 10^−19^	1.000
Soil degradation Weight loss rate	11.101	886.763	4.763730 × 10^−14^	0.992

**Table 8:** Data range of various properties of polyester cotton matrix mycelium composites prepared in this study.

Range of MBCs Performance Data in This Study	
Compressive strength (Mpa)	0.075–0.317
Flexural strength (Mpa)	0.462–0.791
Impact performance score (score)	9–17
Water vapour transmittance (g/m^2^·24 h)	47–112
28 day soil degradation weight loss rate (%)	27–45

**Table 9:** Previous research on mycelium materials and performance data range of traditional materials (adapted from Aiduang et al. [31] and Jones et al. [24]).

Performance Materials	MBCs Previous Studies	PS	PU	PW	PB
Compressive strength (Mpa)	0.25–1.87	0.03–0.69	0.002–48	8–25	1.8–3.4
Flexural strength (Mpa)	0.05–4.4	0.07–0.70	0.21–57	35–78	1.5–7
Impact strength (kJ/m^2^)	0.21–2.70	0.01–0.15	1.0–1.2	–	–
Water absorption (%)	105.07–208.82	0.03–9	0.01–72	5–49	30.1–200
Rate of degradation	Weeks–months	Decades–centuries		Years–decades	

PS = polystyrene, PU = polyurethane, PW = plywood, PB = particle board, “–” = not reported.

**Table 10:** Element extraction and geometric figure transformation.

Type of Element	Inspiration Extraction/Explanation		Geometric Figure Transformation
Architectural landscape	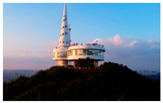	Lianhua Mountain viewing platform	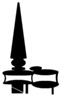
	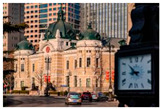	Yokohama Zhengjin Bank Dalian Branch, now Bank of China Zhongshan Branch	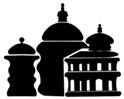
	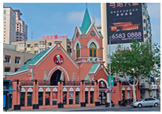	Dalian Christ Church site, now KFC restaurant	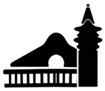
	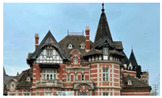	The former site of the East Qing Ship Club, now Dalian Art Museum	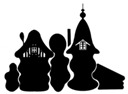
	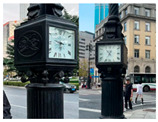	Clock in Zhong Shan Square	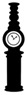
Urban landscape	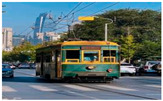	The 201 tram	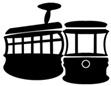
	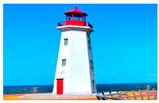	Portia Bay Park Lighthouse	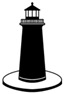
	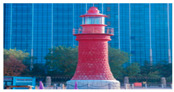	Dalian old wharf lighthouse	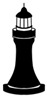

**Table 11:** Experiential cultural and creative evaluation index of mycelium composite materials.

Indicators of Evaluation	Description of Indicators
zb1 saves wood	Maximise the variety and quantity of materials to improve the utilisation rate of materials.
zb2 reused	The ways, methods, means, and forms of waste polyester and cotton material utilisation are rich and diverse
zb3 life cycle	The energy consumption and carbon emission of the mycelium culture process, the degradation efficiency of the product after waste, and whether to reduce the use of traditional chemical materials
zb4 durability	The structure is strong and stable, durable, safe, and reliable
zb5 aesthetics	Product shape, colour, and texture are beautiful
zb6 interactive fun	Whether the user can have fun in the exploration process
zb7 operate	Module combination logic, furnishing’s interspersed structure is simple and easy to understand
zb8 emotional reflection	Through deep thinking about waste textiles and resources, strengthen environmental awareness
zb9 industrialisation	Reasonable cost, whether it can achieve standardised, modular design of mass production

**Table 12:** The experiential product evaluation score of mycelium composite material.

Cultural and Creative Products	zb1	zb2	zb3	zb4	zb5	zb6	zb7	zb8	zb9	Composite Score
Mosaic non-fire aromatherapy box	4.8	4.8	3.9	3.2	4.7	4.8	2.7	4.8	3.9	4.18
Decorative ornaments	3.3	4.3	3.9	3.9	2.8	2.9	4.7	4.8	3.2	3.76
Mosaic decorative painting	3.5	4.2	3.9	4.8	3.2	4	4.6	3.8	4.2	4.02

## Data Availability

Data are contained within the article.
